# Changes in Body Composition, Cardiovascular Disease Risk Factors, and Eating Behavior after an Intensive Lifestyle Intervention with High Volume of Physical Activity in Severely Obese Subjects: A Prospective Clinical Controlled Trial

**DOI:** 10.1155/2013/325464

**Published:** 2013-04-22

**Authors:** Kjersti Karoline Danielsen, Mette Svendsen, Sverre Mæhlum, Jorunn Sundgot-Borgen

**Affiliations:** ^1^Department of Sports Medicine, The Norwegian School of Sport Sciences, P.O. Box 4014 Ullevaal Stadion, 0806 Oslo, Norway; ^2^Department of Endocrinology, Obesity and Preventive Medicine, Oslo University Hospital, P.O. Box 4956 Nydalen, 0424 Oslo, Norway; ^3^NIMI, P.O. Box 3843 Ullevaal Stadion, 0805 Oslo, Norway

## Abstract

We examined the effects of a 10–14-weeks inpatient lifestyle modification program, including minimum 90 min of physical activity (PA) five days/week, on body composition, CVD risk factors, and eating behavior in 139 obese subjects (BMI 42.6 ± 5.2 kg/m^2^). Completion rate was 71% (*n* = 71) in the intensive lifestyle intervention (ILI) group and 85% (*n* = 33) among waiting list controls. Compared to controls body weight (−17.0 (95% CI: −18.7, −15.3) kg, *P* < 0.0001), fat mass (−15.2 (95% CI: −17.4, −13.1) kg, *P* < 0.0001), fat free mass (−1.2 (95% CI: −2.2, −0.2) kg, *P* = 0.016) and visceral fat (−86.6(95% CI: −97.4, −75.7) cm^2^, *P* < 0.0001) were reduced in the ILI-group after 10–14 weeks. Within the ILI-group weight loss was −23.8 (95% CI: −25.9, −21.7) kg, *P* < 0.0001 and -20.3 (95% CI: −23.3, −17.3) kg, *P* < 0.0001, after six and 12 months, respectively. Systolic BP, glucose, triglycerides, and LDL-C were reduced, and HDL-C was increased (all *P* ≤ 0.006) after 10–14 weeks within the ILI group. The reduction in glucose and increase in HDL-C were sustained after 12 months (all *P* < 0.0001). After one year, weight loss was related to increased cognitive restraint and decreased uncontrolled eating (all *P* < 0.05). Thus, ILI including high volume of PA resulted in weight loss with almost maintenance of fat-free mass, favorable changes in CVD risk factors, and eating behavior in subjects with severe obesity.

## 1. Introduction 

Severe obesity (SO: BMI ≥ 40 kg/m^2^ or BMI 35–39.9 kg/m^2^combined with at least one weight-related comorbidity) is associated with a high prevalence of comorbidities as well as high mortality rates and represents a significant public health concern [1–3]. The main goals in the treatment of SO are to reduce comorbidity and mortality. Reduction of fat mass (FM), especially visceral fat area (VFA), and maintenance of fat-free mass (FFM) may lead to improvements in obesity-related cardiovascular disease (CVD) risk factors [4] and minimize the decrease in resting energy expenditure (REE) following weight loss [5–9].

In subjects with a BMI < 35 kg/m^2^ physical activity (PA) and cardiorespiratory fitness have been shown to reduce morbidity and CVD mortality [10–13]. Despite minimal weight loss, favorable changes in body composition (BC) by PA have been demonstrated [14–16], and increased PA is recommended for weight loss maintenance [17, 18]. The mechanisms for the influence of PA on weight loss maintenance are not fully understood, but in addition to PA affecting weight directly through energy expenditure, increased sensitivity for satiety signals and increased inhibitory control of the drive to eat may be involved [19]. However, subjects with SO often experience difficulties with PA due to increased body weight (BW), reduced mobility, and medical problems [20–23]. Some subjects with SO therefore feel that surgery-induced weight loss is necessary for them to increase their PA level [21]. In line with this, it has been claimed that exercise programs may be unhelpful in the treatment of subjects with SO [24]. On the other side, subjects with SO have also reported that they like being physically active and have positive experiences with adapted PA [20–22]. Exercising together with other SO subjects and being introduced to adapted exercises and special equipment may be of great importance in order for this population to increase compliance in PA interventions [21, 23]. 

Both day and inpatient lifestyle interventions cause clinically meaningful weight loss (≥5–10%) the first year after treatment of patients with SO [25–30]. However, data on changes in BC after an inpatient intervention are sparse [31]. Also, none of the previously published studies have included a nontreated control group. One advantage of inpatient treatment, with all PA being mandatory, is that the PA level can more easily be controlled, and attendance can be used as a measure of compliance. 

The primary aim of this nonrandomized clinical controlled trial was to examine the effects of a 10–14-week inpatient lifestyle modification program, including minimum 90 min of PA five days per week, on BC: BW, FM, FFM, and VFA in SO men and women. Secondly, we wanted to investigate changes in CVD risk factors within the intervention group and also observe whether changes in BC and CVD risk factors were maintained at six- and 12-month followup from baseline. Moreover, we studied the possible influence of the intensive lifestyle intervention on appetite regulation by an assessment of eating behavior.

## 2. Materials and Methods 

### 2.1. Study Design

**Figure 1:**
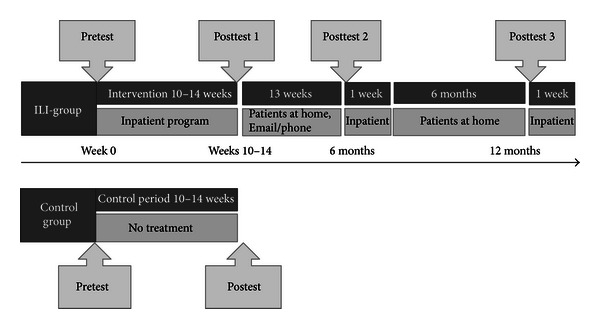
The schedule of the nonrandomised controlled clinical trial and the prospective follow-up study; participant recruitment, assessments, and stays during the lifestyle programme at NIMI Ringerike.

This study was conducted from September 2010 to March 2012. The first part of the study was a nonrandomized controlled clinical trial. The intervention was a predefined comprehensive inpatient lifestyle intervention (ILI) for the treatment of men and women with SO provided by The NIMI Ringerike obesity clinic in Norway. A control group with subjects referred to the clinic, but still on waiting list to start the treatment program, was included in the study, and the group was followed for a time period comparable to the intervention. The controls received no treatment from the clinic during the intervention period. In the second part we prospectively followed the subjects in the ILI group for a one-year period. The design and flow schedule of the study are shown in Figure 1. The program consisted of one 14-weeks inpatient program, which was shortened to 10 weeks during the study. The subjects were then discharged and returned to the clinic for one week of inpatient followup at approximately six and 12 months after the start of the inpatient program. Data were collected at the start (week 0) and the end (weeks 10–14) of the main inpatient treatment program, and at the two follow-up weeks (six and 12 months). The Regional Committee for Medical and Health Research Ethics in Norway approved the protocol, and all the participants provided written informed consent. The study meets the standards of the Declaration of Helsinki and is registered in the ClinicalTrials.gov-registry under the unique trial number NCT01675713.

### 2.2. Study Sample

**Figure 2:**
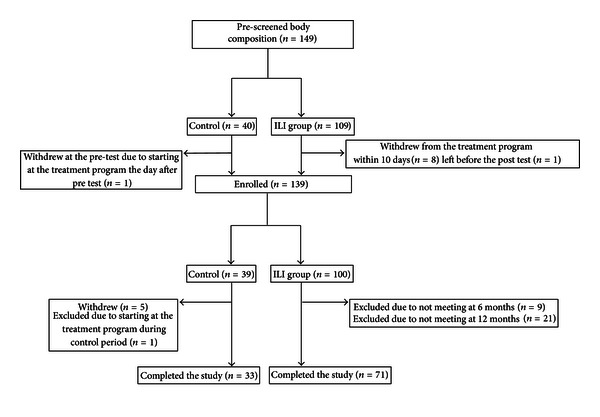
Flow of participants throughout the study.

The study sample consisted of patients referred to the clinic from tertiary care obesity centers when weight loss of clinical importance was not achieved in less-intensive treatment programs in primary or secondary care. The inclusion criteria for participation in the treatment program were BMI ≥ 40 kg/m^2^ or BMI ≥ 35 kg/m^2^ with comorbidities at the time of referring, age between 18 and 65 years, and that the subjects had to be able to walk slowly for approximately 20 minutes. Some subjects left the treatment within the first few days. Hence to be enrolled in the study, participants in the ILI group had to fulfill the main inpatient treatment program. The controls were continuously recruited as they were referred to the waiting list. Exclusion criteria for enrollment in the study were pregnancy or participation in previous treatment programs at the clinic. Of the 149 subjects eligible for the study, a total of 139 (100 in the ILI-group and 39 in the control group) were enrolled. A flow chart of the participants is shown in Figure 2. The completion rate was 71% (*n* = 71) in the ILI-group and 85% (*n* = 33) in the control group.

### 2.3. Intervention

#### 2.3.1. Main Inpatient Treatment Program

A multidisciplinary team including a medical doctor, a psychologist, a clinical nutritionist, nurses, exercise scientists, and physiotherapists provided the ILI. During the 10–14-week inpatient program the subjects were living at the clinic. From week 4 the subjects were allowed to go home for the weekends. All subjects received the same inpatient intensive lifestyle modification program focusing on increasing the PA level, adjusting energy and nutrition intake, and learning coping strategies. Details regarding the program used in this study have been published previously [32]. Both theoretical and practical lessons were provided. During the inpatient periods the subjects participated in two to three mandatory training sessions during the weekdays, each lasting for a minimum of 45 min and supervised by exercise scientists and physiotherapists. Aerobic, strength, agility, and balance training were all included in the training program, as well as different sport activities, games, walking tour, and a day trip. Duration and intensity of the training sessions were gradually progressed. In addition most participants conducted training sessions (e.g., brisk walking) on their own before breakfast and in the evenings, and they were encouraged to work out in the weekends. Regarding dietary intake breakfast and lunch were served as buffets and dinner at plates, whereas the three between-meal snacks were free options of fruit, vegetables, or yoghurt. Portion sizes according to restricted energy levels (1900 kcal/d for men and 1600 kcal/d for women) were demonstrated in the eating room by nutritionists. The macronutrient composition of the diet was planned to be <30 E% from fat, ~20 E% from protein, and 50–60 E% from carbohydrates. In addition the subjects were offered a minimum of 2 × 30 min individual dietary nutritional consultations. Moreover, all subjects attended group session including instructions and practice in methods for behavior modification and coping strategies. The purpose of the course was to help subjects change and sustain their change of lifestyle after finishing the program after hospitalization. The methodological elements were short-term goal setting with specific tasks (in physical activity and nutrition), problem solving, and evaluation. The subjects were also offered individual consultations with a psychologist when needed.

#### 2.3.2. Followup

Followup between the main inpatient program and six months was not structured and was of individual choice (phone calls or e-mail communications). No formal followup between six and 12 months was provided. During the two inpatient follow-up weeks after six and 12 months reminders of coping techniques were given, and the subjects' motivation for exercise and healthy eating was refreshed. 

### 2.4. Measurements

#### 2.4.1. Body Composition

All anthropometric measures were assessed with subjects wearing light clothing without shoes. BC was measured using direct segmental multifrequency (DSM) bioelectrical impedance (Inbody 720, Body Composition Analyzer, Biospace Co. Ltd.), after standardized procedures including a minimum of 2-hour fasting and no drinking or hard training prior to the test. The eight-polar BIA Inbody used in the present study is shown to offer accurate estimates of total body water and extracellular water in SO women with a BMI up to 48.2 kg/m^2^ [33]. Repeated measurements were conducted at approximately the same time of the day for each subject. Height was measured in a standing position at baseline, and this measure was used to calculate BMI (kg/m^2^).

#### 2.4.2. Cardiovascular Disease Risk Factors

Blood samples were collected between 7 and 8 a.m. after an overnight fast and analyzed for triglycerides (TG), serum glucose, total, low-density lipoprotein (LDL), and high-density lipoprotein (HDL) cholesterol (mmol/L) using standard laboratory techniques at Ringerike hospital; Vestre Viken Hospital Trust, 3004 Drammen, Norway. Blood pressure (BP: mmHg) was measured once in sitting position after 5–10-minute rest, using the Reister, big ben round apparatus, cuff size large (arm size 33–41). A medical doctor registered the use of blood pressure-, lipid-, or glucose-lowering medication at baseline. Metabolic syndrome was defined according to the modified ATP III criteria [34]. Waist circumference (WC) was measured at the level of the narrowest point between the lower costal (10th rib) border and the iliac crest [35]. Only subjects with measurement of all the five diagnosis criteria of the metabolic syndrome (i.e., elevated WC, TG, BP, fasting glucose, and reduced HDL) at all four measurement times were included in the analyses (*n* = 52). The use of medication was only recorded at baseline; therefore subjects taking antihypertensive or blood glucose reducing pharmacotherapy were defined with elevated BP and fasting glucose, respectively, in the categorization of metabolic syndrome at all four measurement times. 

#### 2.4.3. Eating Behavior

The 21-item Three-Factor Eating Questionnaire (TFEQ-R21) was used in the assessment of eating behavior. The TFEQ-21 comprises 21 items and covers three eating behavior scales ranging from 0 to 100: cognitive restraint (CR: the tendency to control food intake in order to influence body weight and body shape), uncontrolled eating (UE: the tendency to lose control over eating when feeling hungry or when exposed to external stimuli) and emotional eating (EE: the propensity to overeat in relation to negative mood states, e.g., when feeling lonely, anxious, or depressed). Higher scores indicate more restraint, uncontrolled, and emotional eating [36].

#### 2.4.4. Exercise Capacity

As a measure of compliance, changes in exercise capacity were monitored within the ILI-group using the 10-metre shuttle run test [37, 38] and time spent on a 750-metre walk/run test before and after the 10–14-week inpatient period.

### 2.5. Statistical Analysis

Data are reported as mean (standard deviation), or (95% CI), or number (%). Independent samples *t*-tests, Mann-Whitney *U* test, and Chi-square were used as appropriate to compare groups. Likewise, paired samples *t*-tests and Wilcoxon rank tests were used to assess changes within the ILI group. All tests were two sided, and statistical significance was accepted when *P* < 0.05. There was little change in the *P* values, and hence there was no change in the conclusion, using nonparametric tests. Data are therefor reported as mean (standard deviation), or (95% CI), or number (%), and *P* values from parametric tests unless stated otherwise. Repeated measurement general linear model (GLM) was used to assess within group changes in CVD risk factors, with adjustment for gender, age, and use of relevant medicine (i.e., blood pressure-, lipid-, or glucose-lowering medication) at baseline, whereas Cochran's *Q* test was used to assess change in the proportion of participants diagnosed with metabolic syndrome across the four measurement time points: week 0, weeks 10–14, six, and 12 months. Only subjects with measurement at all four measurement times were included in the analysis for each variable. Regression analysis was performed using univariate GLM; changes in weight were used as dependent variables, and changes in eating behavior as independent variables. Subjects with measurement at the two measurement times were included in the analysis for each change period. Drop-out analysis within the ILI-group showed no significant differences in any of the baseline characteristics between the completers and noncompleters, or in weight reduction from week 0 to week 10–14 (data not shown). However, subjects who dropped out during followup had a smaller BW reduction than the completers between weeks 10–14 and six months (−3.8 (95% CI: −6.2 to −1.3) kg, *P* = 0.003). Data were analyzed using SPSS software version 18 (SPSS Inc, Chicago, IL, USA).

#### 2.5.1. Power Analysis

Based on the results from the treatment group a priori power analysis was performed to determine the sample size of the control group to detect differences between the groups. The acute weight loss for the treatment group was ~17 kg (13.5%), and with *α* fixed level at 0.05 and a power set at 0.80 a minimum of 40 participants in the control group would be required to investigate both 5% and 10% weight reduction in the ILI-group compared to the control group, allowing for a 3.5 kg weight loss in the control group due to the placebo effect/changing in lifestyle on their own.

## 3. Results 

### 3.1. Baseline Characteristics

Baseline characteristics of the participants who completed the study are shown in Table 1. The ILI-group was older than the control group, but no other significant differences between the two study groups were seen at baseline. 

### 3.2. Between-Group Changes in Body Composition

**Figure 3:**
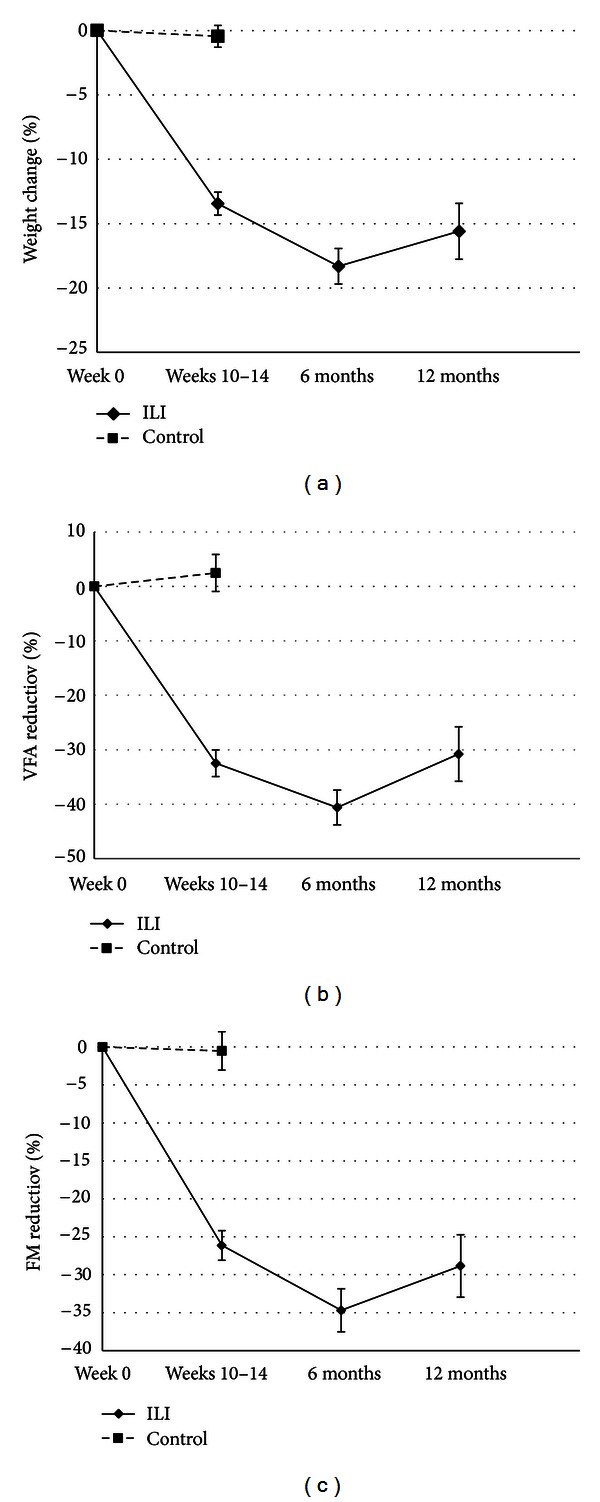
(a) Mean (95% CI) percentage weight loss during followup. (b) Mean (95% CI) percentage reduction in VFA during followup. (c) Mean (95% CI) percentage reduction in FM during followup.

Compared to the control group, reductions in all BC variables were seen in the ILI-group from baseline to weeks 10–14 (Table 2 and Figure 3).

### 3.3. Changes in Weight and Body Composition within the ILI-Group

Changes in BC within the ILI-group at weeks 10–14, six, and 12 months are shown in Table 3. After six- and 12-month followup BW reduction was −23.8 ((95% CI: −25.9, −21.7) kg, *P* < 0.0001) and −20.3 ((95% CI: −23.3, −17.3) kg, *P* < 0.0001), respectively, compared with baseline. Within the ILI-group, the mean (SD) percentage weight loss at 12 months was 15.6% (9.2%), and VFA and FM were reduced by 30.8% (21.1%) and 28.8% (17.4%) compared to baseline, respectively, (Figure 3). 

The proportions of subjects in the ILI-group achieving ≥5%, 10%, 15%, and 20% reduction of their initial weight, respectively, are shown in Table 4. 

**Figure 4:**
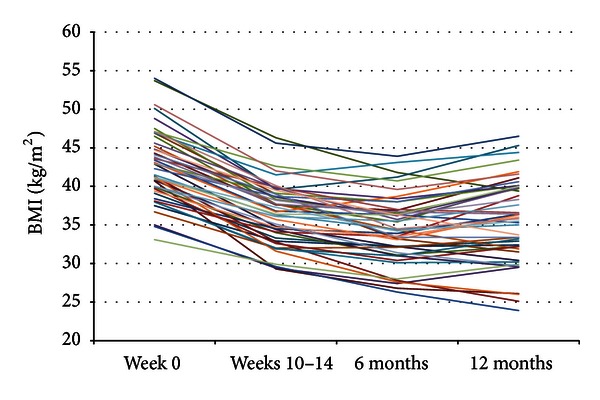
BMI value for each subject within the ILI-group at week 0, week, 10–14, six, and 12 months. Note: two subjects had a BMI < 35 kg/m^2^ at week 0 due to a small weight reduction between the time of referring and the start of the treatment program.

Figure 4 shows the development in BMI of each subject in the ILI-group. Fifty-two (73.2%) of the subjects had BMI ≥ 40.0 kg/m^2^ at week 0, and 51 (71.8%) had BMI < 40.0 kg/m^2^ at 12-month followup.

### 3.4. Changes in Cardiovascular Disease Risk Factors, Metabolic Syndrome, and Exercise Capacity within the ILI-Group

**Figure 5:**
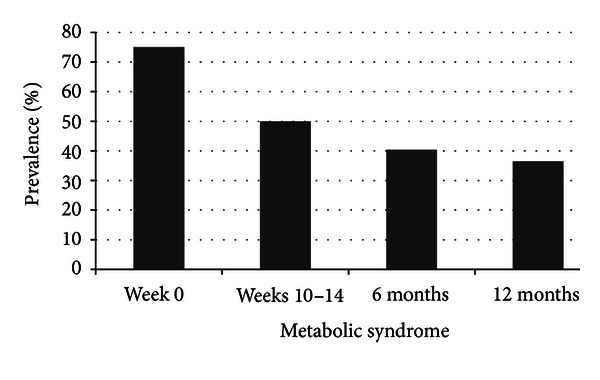
The prevalence of metabolic syndrome within the ILI-group at week 0, weeks 10–14, six, and 12 months (*n* = 52). *P* < 0.0001 for change in the proportion of participants diagnosed with metabolic syndrome across the four measurement time points (Cochran's *Q* test).

At baseline a total of 27 (38.0%), eight (11.3%), and six (8.5%) in the ILI-group were using blood pressure-, lipid- and glucose-lowering medication, respectively. Changes in CVD risk factors and exercise capacity within the ILI-group are shown in Tables 5 and 6. The prevalence of metabolic syndrome was reduced significantly (*P* < 0.0001) within the ILI-group across the four time points (Figure 5).

### 3.5. Eating Behavior before and after Intervention

**Figure 6:**
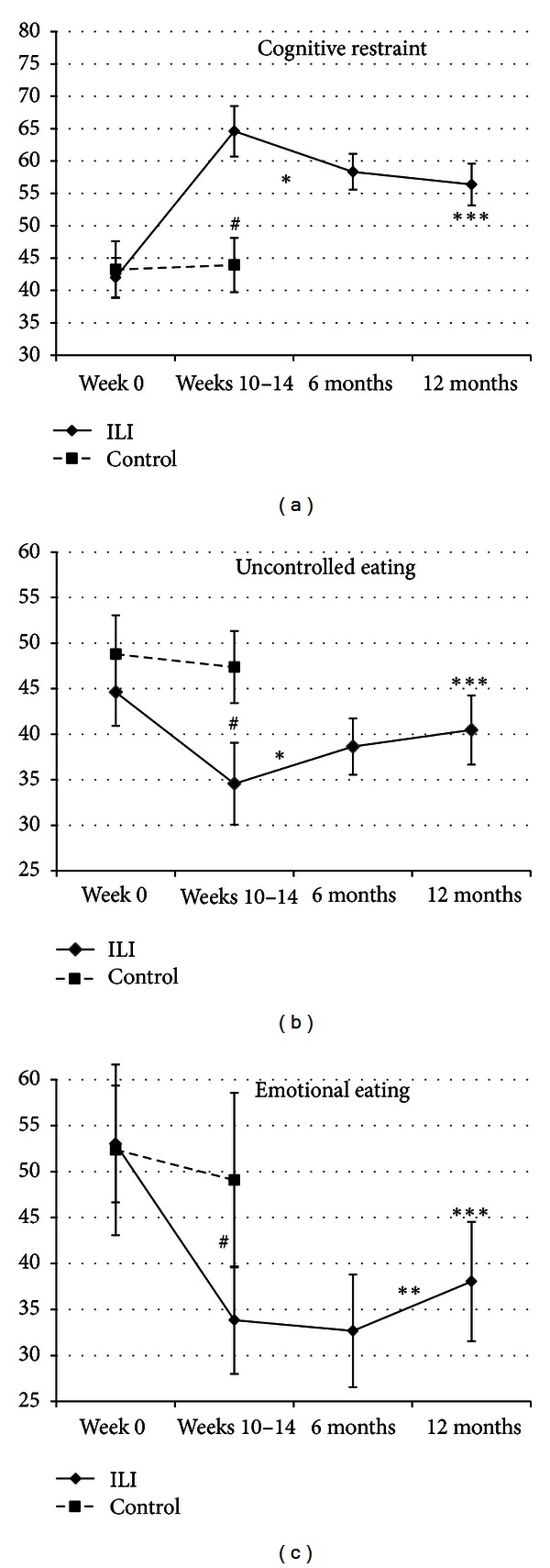
Mean (95% CI) scores of eating behavior (TFEQ-21) at week 0, weeks 10–14, six, and 12 months followup. ^#^ *P* < 0.007 for differences in changes between ILI-group and controls, *P < 0.02 for changes within the ILI-group from weeks 10–14 to six months, **P = 0.012 for changes within the ILI-group from six to 12 months, and ***P < 0.04 for changes within the ILI-group from week 0 to 12 months.

Group comparisons of scores on the TFEQ-R21 showed no differences in eating behavior at baseline (Table 7). Eating behavior in the ILI-group and controls prior to and after treatment is shown in Figure 6. Together these changes resulted in a significant reduction in UE and EE (*P* = 0.033 and *P* < 0.0001) and increase in CR (*P* < 0.0001) scores from week 0 to 12 months of followup within the ILI-group, where the main changes occurred during the intervention period and remained quite stable thereafter. 

Table 8 shows the univariate GLM analysis with respect to changes in weight and eating behaviour, respectively. 

## 4. Discussion 

The major findings of this study were that men and women with SO reduced FM and VFA, with a minimal reduction in FFM, during an intensive lifestyle intervention including a minimum of 90 minutes of PA five days a week. The main changes in BC occurred during the 10–14 weeks of the intervention period. The changes remained quite stable during followup and almost 50% achieved ≥15% weight reduction at 12 months. 

We found that BW decreased by 1.5 kg per week during the intervention. This is in line with other studies investigating inpatient treatment programs for SO subjects [25, 31] but greater than reported following outpatient treatment programs [25, 27, 28]. The important finding in this study was that FFM was almost maintained during weight loss. In the present study the minimum amount of PA was 90 minutes five days per week and the reduction in FFM accounted for 10% of total weight loss during the intervention period. The amount of PA was higher in our study as compared to the five training sessions of 30–40 minutes per week reported by Maffiuletti et al. [31] and the 60-minutes sessions five days a week reported by Goodpaster et al. [27]. Loss of FFM constituted 24% and 22% of total BW reduction, respectively, in these studies. In addition, the total loss of BW and FM in our study at six months was twice the reductions reported by Goodpaster et al. However, the reduction in kg FFM was almost the same. Taken collectively it seems that a minimum of 90 minutes of PA five days per week may be necessary during a weight reduction period to maintain FFM in SO subjects.

Maintenance of FFM is of particular importance in obesity treatment in order to minimize the reduction in energy expenditure seen after weight loss [5, 7–9, 39–41]. Skeletal muscle mass (SMM) was reduced by about 0.1 kg per week during the intervention period and accounted for 71% of the reduction in FFM. Despite the strength training included in our ILI, no increase in SMM was observed. This may be due to the negative energy balance [42, 43], lower BW and hence less stimulation for muscle growth in the lower limbs. However, an increase in muscle growth was not the aim of the strength training program/exercises used in our intervention program.

In the present study, a decrease of 30% in VFA was achieved at weeks 10–14 and maintained at 12 months. This might be explained by the high volume of exercise included in our study, as a dose-response relationship between amount of exercise and the improvements in central obesity has been shown in less obese subjects [15, 44]. A significant reduction in the prevalence of metabolic syndrome was observed within the ILI-group during the study. Furthermore we observed significant favorable changes in most CVD risk factors following the inpatient period; the exception being diastolic BP and total cholesterol. However, we found an increase in HDL cholesterol and a decrease in the level of glucose, changes that remained significant at 12 months. This is in accordance with the increase in HDL cholesterol seen in SO subjects reporting PA energy expenditure of 450 kcal min per day after a mean weight loss of 45 kg [45]. Thus the high level of PA may be partly responsible for the favourable changes in these metabolic risk factors. The observed lack of sustained improvements in the other CVD risk factors has also been reported by others [27, 31] and may be due to low baseline comorbidity. Moreover, changes in the use of medication may also have influenced the results as supported by the decrease in systolic and diastolic BP seen at 12-month follow up among the subjects not using BP-lowering medication. 

PA is associated with weight loss and increased cardiorespiratory fitness in subjects with SO undergoing lifestyle treatment [29]. In addition to the well-known effect of PA on body weight through energy expenditure, different research lines also suggest an indirect effect through the positive influence of PA on appetite regulation and eating behavior [19, 46–51]. Increasing restrictive eating and decreasing uncontrolled and emotional eating are important for weight maintenance after weight loss [47, 51, 52]. In the present study we found that increased cognitive restraint and decreased uncontrolled eating were related to weight loss after one year. Indeed, it has been shown that PA has positive influence on appetite regulation by increased sensitivity for satiety signals and improvements in eating behaviors [19, 46, 47, 49–51]. We speculate that the severely obese subjects in the present study by increasing PA also increase their cognitive restraint by a spill-over effects as shown by others [47, 49].

The present study has some strengths and limitations. The main strength was the mandatory PA program in an inpatient setting. We also measured BC and were able to assess the changes in BC in a weight loss intervention with high volume of PA. The main limitations of the study were that we did not objectively measure PA level or energy intake, and hence we cannot determine how much of the weight loss and improvements in health risk to be attributed to altered caloric restriction, change in nutrient composition, or exercise, but we have examined the effect of an lifestyle intervention program as a whole. Another limitation of our study was that we did not measure cardiorespiratory fitness and appetite-related hormones. However, results from the walking tests and TFEQ indicated improved fitness and eating habits. Finally we have described changes in BC and CVD risk factors for a relatively short time period; whether the results achieved will persist in the long term remains to be seen. There is also a need for studies of mechanisms regarding the influence of PA on appetite regulation.

## 5. Conclusion

This study shows that weight loss achieved by an intensive inpatient lifestyle intervention with high volume of PA resulted in an almost maintenance of FFM and favorable changes in CVD risk factors and eating behavior in subjects with SO. The mean reduction of VFA was 30% one year after the start of the intervention and may partly explain the changes seen in the metabolic risk factors. After one year, about 50% of severely obese subjects maintained more than 15% weight loss. Moreover, the weight loss was related to increased cognitive restraint and decreased uncontrolled eating behavior. 

## Figures and Tables

**Table 1:** Characteristics of participants and body composition at baseline in the intensive lifestyle intervention (ILI) group and control group. Data are given as mean values (standard deviation) or number of subjects and percentages.

	ILI (*n* = 71)		Control (*n* = 33)		*P* value
	*N*	%	*N*	%	
Female/male	42/29	59.2/40.8	21/12	63.6/36.4	0.663^a^
Age (year)	45.2 (9.5)		38.5 (9.8)		0.001^b^
Height (cm)	173.2 (8.6)		172.3 (10.4)		0.626^b^
Body mass index (kg/m^2^)	42.8 (4.6)		42.8 (6.3)		0.983^b^
Body weight (kg)	128.9 (19.4)		127.1 (21.6)		0.680^b^
Fat mass (kg)	60.5 (11.0)		60.4 (13.5)		0.986^b^
Fat-free mass (kg)	68.3 (13.2)		67.1 (12.8)		0.676^b^
Skeletal muscle mass (kg)	38.4 (7.9)		37.9 (7.4)		0.760^b^
Visceral fat area (cm^2^)	255.7 (64.7)		245.6 (66.3)		0.464^b^
Highest education					
Primary and/or secondary school	8^c^	12.9	7	21.2	0.178^a^
High school	32^c^	51.6	20	60.6	
Higher education	22^c^	35.5	6	18.2	
Employment (full-time job)	34^d^	52.3	16^e^	50.0	0.831^a^
Current smoker (yes)	15^f^	23.4	6	18.2	0.552^a^

^a^Chi-square; ^b^independent-samples *t* test; ^c^ *n* = 62, ^d^ *n* = 65, ^e^ *n* = 32, ^f^ *n* = 64.

**Table 2:** Between-group differences in changes in body composition from week 0 to weeks 10–14. Data are mean values (standard deviation or 95% CI).

	ILI (*n* = 71)	Control (*n* = 33)	Between-group differences, mean (95% CI)	*P* value^a^
Body mass index (kg/m^2^)	−5.8 (1.8)	−0.2 (1.0)	−5.6 (−6.2, −5.1)	<0.0001
Body weight (kg)	−17.5 (6.1)	−0.5 (2.8)	−17.0 (−18.7, −15.3)	<0.0001
Fat mass (kg)	−15.7 (5.3)	−0.4 (5.0)	−15.2 (−17.4, −13.1)	<0.0001
Fat-free mass (kg)	−1.7 (2.5)	−0.5 (2.1)	−1.2 (−2.2, −0.2)	0.016
Skeletal muscle mass (kg)	−1.2 (1.5)	0.2 (3.6)	−1.4 (−2.4, −0.4)	0.005
Visceral fat area (cm^2^)	−82.3 (31.6)	4.3 (22.7)	−86.6 (−97.4, −75.7)	<0.0001

^a^Independent samples *t*-test.

**Table 3:** Change in body composition from week 0 to 12 months within the ILI group (*n* = 71).

	Mean change (95% CI) Week 0 to 12 months	*P* value^a^	Mean change (95% CI) Week 0 to weeks 10–14	*P* value^a^	Mean change (95% CI) Weeks 10–14 to six months	*P* value^a^	Mean change (95% CI) Six to 12 months	*P* value^a^
Body mass index (kg/m^2^)	−6.7 (−7.6, −5.7)	<0.0001	−5.8 (−6.2, −5.4)	<0.0001	−2.1 (−2.5, −1.7)	<0.0001	1.2 (0.7, 1.7)	<0.0001
Body weight (kg)	−20.3 (−23.3, −17.3)	<0.0001	−17.5 (−18.9, −16.1)	<0.0001	−6.3 (−7.5, −5.1)	<0.0001	3.5 (2.0, 5.1)	<0.0001
Fat mass (kg)	−17.5 (−20.1, −14.9)	<0.0001	−15.7 (−17.0, −14.4)	<0.0001	−5.2 (−6.2, −4.1)	<0.0001	3.4 (2.1, 4.7)	<0.0001
Fat-free mass (kg)	−2.8 (−3.6, −2.0)	<0.0001	−1.7 (−2.3, −1.2)	<0.0001	−1.1 (−1.5, −0.7)	<0.0001	0.1 (−0.3, 0.6)	0.585
Skeletal muscle mass (kg)	−1.7 (−2.2, −1.3)	<0.0001	−1.2 (−1.6, −0.9)	<0.0001	−0.7 (−0.9, −0.5)	<0.0001	0.2 (−0.1, 0.5)	0.225
Visceral fat area (cm^2^)	−81.0 (−95.3, −66.7)	<0.0001	−82.3 (−89.8, −74.8)	<0.0001	−22.0 (−28.1, −15.9)	<0.0001	23.3 (15.5, 31.1)	<0.0001

^a^Paired samples *t*-test.

**Table 4:** Subjects in the ILI-group achieving ≥5%, 10%, 15%, and 20% weight loss from initial weight at weeks 10–14, six, and 12 months. Data are number of subjects (percentage).

	Weeks 10–14	Six months	12 months
≥5% weight loss	71 (100.0%)	71 (100.0%)	65 (91.5%)
≥10% weight loss	57 (80.3%)	67 (94.4%)	48 (67.6%)
≥15% weight loss	20 (28.2%)	21 (29.6%)	35 (49.3%)
≥20% weight loss	4 (5.6%)	27 (38.0%)	19 (26.8%)

**Table 5:** Change in values for cardiovascular disease risk factors within the ILI-group. Data are mean values (SD or 95% CI).

	Mean Week 0	Mean change Week 0 to 12 months	*P* value	Mean change Week 0 to weeks 10–14	*P* value	Mean change Weeks 10–14 to six months	*P* value	Mean change Six to 12 months	*P* value
s-BP (mmHg)									
All (*n* = 57)	132.3 (10.7)	−2.8 (−6.0, 0.4)	0.082^a^	−6.4 (−9.1, −3.7)	<0.0001^a^	1.1 (−1.7, 4.0)	0.431^a^	2.5 (−0.5, 5.4)	0.097^a^
No blood pressure lowering (*n* = 37)	130.4 (8.8)	−3.8 (−6.9, −0.7)	0.018^b^	−5.4 (−8.4, −2.4)	0.001^b^	0.3 (−3.2, 3.8)	0.877^b^	1.4 (−1.7, 4.4)	0.368^b^
d-BT (mmHg)									
All (*n* = 57)	83.7 (6.1)	−1.8 (−4.0, 0.3)	0.094^a^	−1.3 (−3.2, 0.6)	0.175^a^	−2.0 (−4.3, 0.3)	0.088^a^	1.5 (−0.7, 3.7)	0.173^a^
No blood pressure lowering (*n* = 37)	83.2 (5.4)	−3.5 (−6.3, −0.7)	0.015^b^	−2.8 (−5.0, −0.7)	0.012^b^	−2.3 (−5.2, 0.6)	0.112^b^	1.6 (−1.2, 4.4)	0.245^b^
GLUC (mmol/L)									
All (*n* = 58)	5.8 (1.3)	−0.5 (−0.7, −0.3)	<0.0001^a^	−0.7 (−0.9, −0.5)	<0.0001^a^	0.1 (−0.1, 0.2)	0.258^a^	0.1 (0.0, 0.3)	0.150^a^
No glucose lowering (*n* = 54)	5.6 (0.8)	−0.4 (−0.6, −0.2)	<0.0001^b^	−0.6 (−0.8, −0.4)	<0.0001^b^	0.1 (−0.1, 0.2)	0.260^b^	0.1 (−0.1, 0.2)	0.300^b^
HDL (mmol/L)									
All (*n* = 60)	1.1 (0.3)	0.3 (0.3, 0.4)	<0.0001^a^	0.1 (0.0, 0.1)	0.001^a^	0.2 (0.1, 0.2)	<0.0001^a^	0.1 (0.0, 0.2)	0.001^a^
No lipid lowering (*n* = 52)	1.1 (0.3)	0.3 (0.3, 0.4)	<0.0001^b^	0.1 (0.0, 0.1)	0.002^b^	0.2 (0.1, 0.2)	<0.0001^b^	0.1 (0.0, 0.2)	0.004^b^
LDL (mmol/L)									
All (*n* = 59)	2.9 (0.9)	0.4 (0.2, 0.6)	<0.0001^a^	−0.2 (−0.3, −0.1)	0.005^a^	0.3 (0.2, 0.4)	<0.0001^a^	0.3 (0.2, 0.4)	<0.0001^a^
No lipid lowering (*n* = 51)	3.1 (0.8)	0.3 (0.1, 0.5)	0.002^b^	−0.3 (−0.4, −0.1)	<0.0001^b^	0.2 (0.1, 0.3)	<0.0001^b^	0.3 (0.2, 0.5)	<0.0001^b^
TG (mmol/L)									
All (*n* = 59)	1.4 (0.6)	−0.1 (−0.2, 0.1)	0.231^a^	−0.2 (−0.3, −0.2)	<0.0001^a^	0.0 (0.0, 0.2)	0.066^a^	0.1 (−0.1, 0.2)	0.420^a^
No lipid lowering (*n* = 51)	1.4 (0.6)	−0.1 (−0.3, 0.1)	0.185^b^	−0.2 (−0.3, −0.2)	<0.0001^b^	0.0 (−0.1, 0.1)	0.451^b^	0.1 (0.0, 0.3)	0.126^b^
Tot C (mmol/L)									
All (*n* = 60)	4.7 (1.1)	0.5 (0.3, 0.7)	<0.0001^a^	−0.1 (−0.3, 0.0)	0.155^a^	0.4 (0.2, 0.5)	<0.0001^a^	0.3 (0.1, 0.4)	0.002^a^
No lipid lowering (*n* = 52)	4.9 (1.0)	0.4 (0.2, 0.6)	<0.0001^b^	−0.2 (−0.3, 0.0)	0.015^b^	0.3 (0.2, 0.5)	<0.0001^b^	0.3 (0.1, 0.5)	0.002^b^
Waist circumferences (cm)									
All (*n* = 70)	123.5 (12.8)	−14.9 (−16.9, −13.0)	<0.0001^a^	−14.0 (−14.9, −13.0)	<0.0001^a^	−4.6 (−5.6, −3.5)	<0.0001^a^	3.6 (2.5, 4.7)	<0.0001^a^

s-BP: blood pressure systolic, d-BP: blood pressure diastolic, GLUC: fasting glucose, HDL: high-density lipoprotein cholesterol, LDL: low-density lipoprotein cholesterol, TG: triglycerides, Tot C: total cholesterol. ^a^GLM adjusted for age, gender, and relevant medication at baseline (HDL, LDL, Tot C, and TG adjusted for lipid-lowering medication at baseline; s-BP and d-BP adjusted for blood pressure-lowering medication at baseline; and GLUC adjusted for glucose-lowering medication at baseline), ^b^subgroup analysis of participants not using relevant medication at baseline, GLM adjusted for age and gender.

**Table 6:** Change in exercise capacity within the ILI-group. Data are mean values (SD or 95% CI).

	Mean Week 0	Mean change Week 0 to 12 months	*P* value	Mean change Week 0 to weeks 10–14	*P* value	Mean change Weeks 10–14 to six months	*P* value	Mean change Six to 12 months	*P* value
10-metre shuttle run test, exercise time (min) (*n* = 43)	9.3 (2.9)	4.2 (3.3, 5.2)	<0.0001^a^	4.7 (3.9, 5.6)	<0.0001^a^	0.7 (0.3, 1.1)	0.002^a^	−1.2 (−1.6, −0.7)	<0.0001^a^
750-metre walk test, exercise time (min) (*n* = 47)	6.9 (1.0)	−1.4 (−1.6, −1.1)	<0.0001^a^	−1.2 (−1.4, −1.0)	<0.0001^a^	−0.4 (−0.5, −03)	<0.0001^a^	0.2 (0, 0.4)	00.015^a^

^a^Paired samples *t*-test.

**Table 7:** Self-assessment of eating behavior (TFEQ-R21) at baseline in the intensive lifestyle intervention (ILI) group and control group. Data are given as mean values (standard deviation).

	ILI	Control	*P* value^a^
TFEQ CR	42.0 (11.4)^b^	43.2 (12.4)^d^	0.635
TFEQ UE	44.6 (13.6)^c^	48.8 (12.0)^d^	0.150
TFEQ EE	53.0 (23.5)^c^	52.4 (26.2)^d^	0.907

^a^Independent samples *t*-test, ^b^ *n* = 56, ^c^ *n* = 55, ^d^ *n* = 33.

UE: Uncontrolled eating, CR: cognitive restraint, EE: emotional eating.

**Table 8:** Univariate GLM between changes in eating behavior and weight change.

Parameter/independent variable	B	95% CI	*P* value
Regression 1 (*n* = 60): dependent variable weight change week 0 to 12 months			

Change CR week 0 to 12 months	−0.310	−0.501, −0.120	0.002
Change UE week 0 to 12 months	0.252	0.008, 0.496	0.043
Change EE week 0 to 12 months	0.016	−0.135, 0.167	0.831

Regression 2 (*n* = 62): dependent variable weight change week 0 to weeks 10–14			

Change CR week 0 to 10–14	−0.152	−0.230, −0.074	0.000
Change UE week 0 to 10–14	0.064	−0.049, 0.177	0.262
Change EE week 0 to 10–14	0.008	−0.089, 0.073	0.843

Regression 3 (*n* = 62): dependent variable weight change weeks 10–14 to 6 months			

Change CR weeks 10–14 to 6 months	0.005	−0.079, 0.090	0.898
Change UE weeks 10–14 to 6 months	0.011	−0.112, 0.135	0.856
Change EE weeks 10–14 to 6 months	0.087	0.007, 0.168	0.033

Regression 4 (*n* = 61): dependent variable weight change 6 to 12 months			

Change CR week 6 months to 12 months	−0.108	−0.274, 0.057	0.196
Change UE week 6 months to 12 months	−0.019	−0.212, 0.173	0.842
Change EE week 6 months to 12 months	0.030	−0.100, 0.160	0.647
